# A Systematic Review and Meta-Analysis of Randomized Controlled Trials Comparing the Effects of Biguanides (Metformin) and Thiazolidinediones on Glucose Tolerance and Insulin Sensitivity in Patients With Type II Diabetes Mellitus

**DOI:** 10.7759/cureus.39445

**Published:** 2023-05-24

**Authors:** Hany A Zaki, Haris Iftikhar, Nabil A Shallik, Eman Shaban, Nood Dhafi R Al-Marri, Israr Bashir, Awny Elhadad, Fatma Zoghlami, Abeer Abdalrubb

**Affiliations:** 1 Emergency Medicine, Hamad Medical Corporation, Doha, QAT; 2 Anaesthesiology, Weill Cornell Medical College in Qatar, Doha, QAT; 3 Anaesthesiology, Hamad Medical Corporation, Doha, QAT; 4 Cardiology, Al Jufairi Diagnosis And Treatment, Doha, QAT; 5 Emergency Department, United Lincolnshire Hospitals, Lincoln, GBR; 6 Emergency Department, Hamad General Hospital, Qatar, QAT; 7 Endocrinology and Diabetes, Hamad Medical Corporation, Doha, QAT

**Keywords:** diabetes mellitus (t2dm), glucose intolerance, glucose tolerance, insulin resistance, “ metformin” “antidiabetic drugs”, insulin sensitivity, biguanide, thiazolidinedione

## Abstract

Type II diabetes mellitus (T2DM) is a global epidemic affecting people of all ages in developed and developing countries. The disease is usually characterized by insulin resistance and glucose intolerance; therefore, oral antidiabetic drugs such as thiazolidinediones (TZDs) and biguanide metformin are used to counter these defects. Due to the varied action mechanisms of TZDs and Metformin, their effects on insulin sensitivity and glucose tolerance may differ. Therefore, the current study was carried out to compare the effects of Metformin and TZDs on insulin sensitivity and glucose tolerance among patients with T2DM.

Two methods, including using a well-outlined search strategy in 5 electronic databases including ScienceDirect, Google Scholar, PubMed, Scopus, and Embase, and a manual search which involved going through the reference lists of studies from the electronic databases were used to retrieve studies published between 2000 and 2022. Additionally, data analysis of outcomes retrieved from the studies eligible for inclusion and the methodological quality was carried out using the Review Manager software (RevMan 5.4.1) and STATA.

The meta-analysis has shown that TZDs have a significantly better overall effect on fasting plasma glucose (FPG) (SMD:0.61; 95% CI:0.06, 1.16: p = 0.03) and insulin sensitivity than Metformin (Mean QUICKI: 0.306 ± 0.019 vs. 0.316 ± 0.019, respectively; p=0.0003). However, the TZDs and Metformin offer the same effect on glycemic control as assessed using HBA1c levels (MD: 0.10; 95% CI: -0.20, 0.40; p = 0.52).

TZDs offer better insulin sensitivity and glucose tolerance improvements compared to Metformin. This evidence contradicts the current guidelines by the American Diabetes Association/European Association for the Study of Diabetes (ADA/EASD) and the American Association of Clinical Endocrinologists/American College of Endocrinology (AACE/ACE), which recommend the use of Metformin as the first-line drug monotherapy for patients with T2DM.

## Introduction and background

Type II diabetes mellitus (T2DM) is a global epidemic affecting people of all ages in developed and developing countries. As a result of increasing obesity rates and lifestyle changes, the number of people being diagnosed with diabetes is increasing worldwide. Recent statistics by the Centers for Disease Control (CDC) show that in the United States alone, more than 37 million individuals live with diabetes, of which T2DM accounts for approximately 90-95% [1]. This disease is usually more common in people above 45 years; however, it is also becoming more common among children, teens, and young adults. One early and sustained feature of T2DM is insulin resistance (IR), a condition where the body cells fail to respond to normal insulin [2]. When the conditions of IR worsen, the insulin demand increases and a decline in the pancreatic β-cell function allows the glucose tolerance to deteriorate into T2DM. Evidence shows that if the IR is insufficiently compensated by the insulin concentrations, then hyperglycemia supervenes, resulting in extensive microvascular morbidity in patients with T2DM [3,4]. Furthermore, IR is independently associated with cardiovascular risk leading to premature deaths. Therefore, therapeutic strategies to manage insulin resistance and glucose intolerance have been sought to address these issues.

The two classes of oral insulin-sensitizing drugs that have been used to counter insulin resistance are thiazolidinediones (TZDs) which include troglitazone, Rosiglitazone, and pioglitazone) and the biguanide metformin [5]. Metformin was initially licensed as an antihyperglycemic medication in Europe more than 40 years ago when its action mechanism was less known. Since then, Metformin has been used to improve insulin sensitivity among insulin-resistant patients. Metformin usually enhances insulin sensitivity by increasing the insulin-mediated insulin receptor tyrosine kinase activity, activating the post-receptor insulin signaling pathways [6].

On the other hand, TZDs are known to reduce insulin resistance by increasing insulin-dependent glucose disposal and reducing hepatic glucose output [7]. Despite these drugs being used to reduce insulin resistance and glucose tolerance, their effects may differ due to the different action mechanisms. Therefore, this systematic review and meta-analysis were conducted to compare the effects of TZDs and Metformin on insulin sensitivity and glucose tolerance among patients with T2DM.

## Review

Methodology

Protocol and Registration

We prepared this systematic review and meta-analysis according to the Cochrane Collaboration guidelines and results reported per the PRISMA (Preferred Reporting Items for Systematic Review and Meta-Analyses) guidelines. For the protocol and registration of this manuscript, we followed the PROSPERO database protocol and registration.

Eligibility Criteria

Two reviewers assessed the retrieved relevant studies using the inclusion and exclusion criteria. For studies to be included in the current systematic review, they had to meet the following criteria: Articles published in English. The reviewers created this criterion to ensure no direct translation of scientific terms, which could lead to a loss of meaning and context. Studies compared TZDs to Metformin in patients with T2DM, with studies with a sufficient pool of patients, i.e., more than ten patients. This criterion enhanced the current review's scientific research, statistical power, and Studies on Human subjects.

Studies were excluded from the current study based on the following criteria: studies published in languages other than English, studies that evaluated only combination therapies, i.e., TZDs plus metformin or TZDs plus metformin plus insulin, etc., studies that compared either TZDs or Metformin to other drugs, studies that included patients without T2DM, e.g., obese patients, and studies designed as systematic reviews and meta-analyses, letters to the editor, case reports, and abstracts without evidence of entire articles.

Literature Search

An intensive search for articles related to our topic was conducted using two strategies. First, a detailed search strategy that utilized the Boolean expressions "AND" or "OR" to combine the search terms was used in five electronic databases, including ScienceDirect, Google Scholar, PubMed, Scopus, and Embase. This search strategy was as follows; (thiazolidinediones OR rosiglitazone OR troglitazone OR pioglitazone) AND (biguanide OR metformin) AND (insulin sensitivity OR insulin resistance OR insulin action) AND (glucose tolerance OR glucose production OR glucose intolerance) AND (T2DM OR Type II Diabetes mellitus). The other method involved reviewing the reference lists of relevant studies from the electronic databases to acquire more studies for review in the current study. All articles retrieved using these methods must have been published between 2000 and 2022.

Data Extraction

The process of retrieving relevant data from studies eligible for inclusion in the current study was independently performed by three reviewers. The data retrieved by these reviewers included Author ID (The surname of the first author and year of publishment), characteristics of the participants (the sample size, age, and gender), study design, Metformin and TZDs dosages, follow-up period, and the main outcomes. The primary outcomes of our study were insulin sensitivity measured using the Quantitative Insulin Sensitivity Check Index (QUICKI), which is calculated from fasting insulin and glucose using the formula 1/ (log10 FSI ± log10 FBG) [8], and glucose tolerance, which will be assessed through fasting plasma glucose (FPG). The secondary outcome of this systematic review was glycemic control which was evaluated using glycosylated hemoglobin (HBA1c). The inconsistencies in extracted data prompted the three reviewers to debate to reach a consensus. If an agreement was not reached, a fourth reviewer was consulted.

Quality Assessment

**Figure 1:**
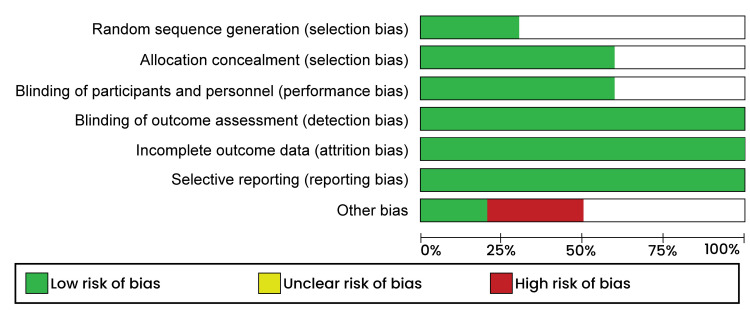
Risk of bias graph

**Figure 2:**
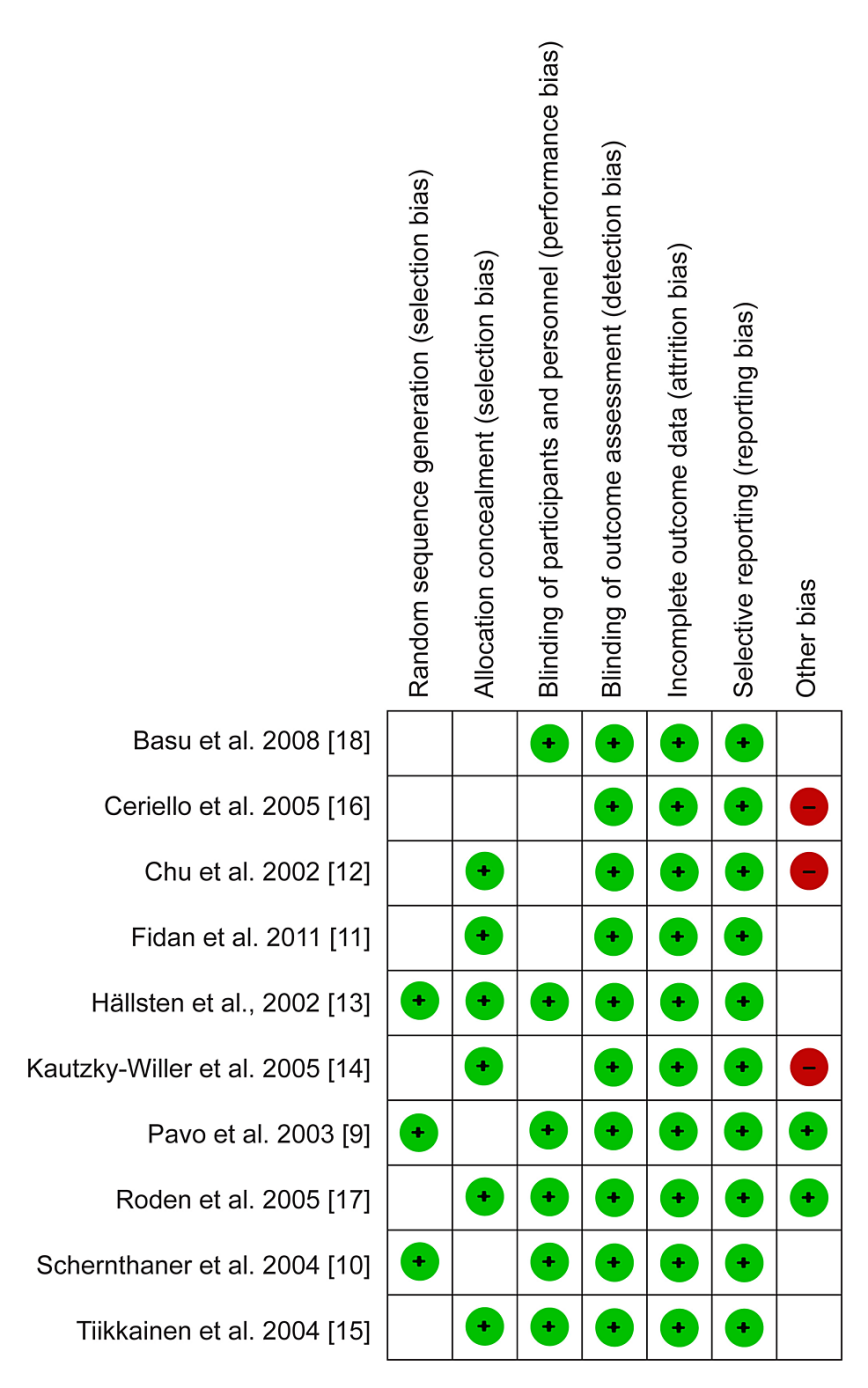
Risk of bias summary Pavo et al.,2003 [9], Schernthaner et al.,2004 [10], Fidan et al.,2011 [11], Chu et al.,2002 [12], Hällsten et al.,2002 [13], Kautzky-Willer et al.,2005 [14], Tiikkainen et al.,2004 [15], Ceriello et al.,2005 [16], Roden et al.,2005 [17], Basu et al.,2008 [18]

The quality appraisal was done using the Risk of Bias tool in the Review Manager software (RevMan 5.4.1). This assessment was carried out by an independent reviewer who then categorized the elements; selection, performance, attrition, and reporting bias in each study into "low risk," "High risk," and "unclear risk." A high risk of bias was used to refer to an insufficiently addressed element, while a low risk of bias meant a particular element was thoroughly discussed. On the other hand, an unclear risk of bias was assigned if the reviewer could not judge any aspect due to fewer details. The risk of bias graph is presented in Figure 1. And bias summary is presented in Figure 2.

Data Synthesis

The pooled effects of TZDs and metformin on glucose tolerance and glycemic control were carried out using the RevMan software. The outcomes related to glucose tolerance were presented as means; therefore, the meta-analysis was carried out using the standard mean difference (SMD). Due to the expected heterogeneity, a random effect model was also selected. This heterogeneity was measured using I2 statistics, of which heterogeneity values of 0 - 49%, 50 - 70%, and above 70% were considered low, moderate, and high, respectively. A 95% confidence interval was also employed to improve our meta-analysis’s statistical power, and the statistical difference was explained by p < 0.05. A statistical analysis using STATA software was also conducted to compare the effect of TZDs and metformin on insulin sensitivity. All meta-analyses were then presented in forest plots, while the results of the statistical analyses were presented in tabular form.

Results

Search Results

**Figure 3:**
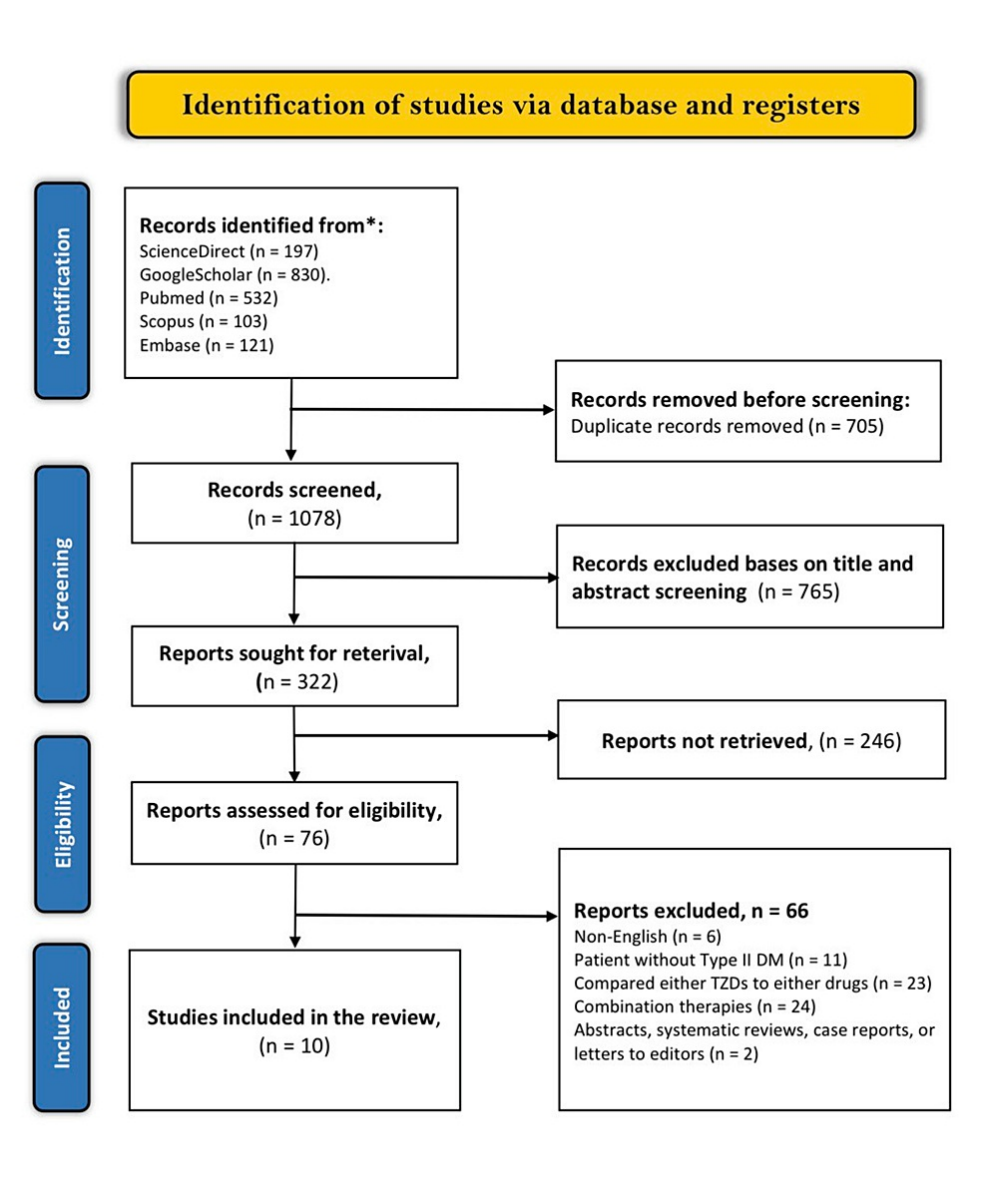
PRISMA flow diagram of the literature search results PRISMA: Preferred Reporting Items for Systematic Reviews and Meta-Analyses

The literature search through the mentioned electronic databases yielded 1783 articles related to our study. A detailed screening process of the 1783 articles led to the exclusion of 705 duplicates. The other 1078 articles then had their titles and abstracts screened, of which only 322 met the screening criteria. Of the remaining 322 articles, 246 were not retrieved, and the other 76 articles were assessed using the eligibility criteria. This assessment led to the inclusion of 10 articles while the other 66 articles were excluded as follows; 6 were non-English articles, 11 included patients without T2DM, 23 either compared TZDs or metformin to other drugs, 24 evaluated combination therapies and 2 were systematic review and meta-analyses. The study selection results are presented in the PRISMA flow diagram below (figure 3). Characteristics of included studies are presented in (Table 1). 

**Table 1:** Study characteristics Pavo et al.,2003 [9], Schernthaner et al.,2004 [10], Fidan et al.,2011 [11], Chu et al.,2002 [12], Hällsten et al.,2002 [13], Kautzky-Willer et al.,2005 [14], Tiikkainen et al.,2004 [15], Ceriello et al.,2005 [16], Roden et al.,2005 [17], Basu et al.,2008 [18]

Author ID	Study Design	Participant characteristics	Metformin	TZDs	Follow-up (weeks)	Main outcomes
Pavo et al.,2003 [9]	RCT	205 patients (103 females and 102 males, aged at least 40 years)	100 patients in the metformin group received a mean dosage of 2292 mg/d	105 patients in the pioglitazone group received a mean dosage of 41.5 mg/d	32	The patients in the pioglitazone group had a more pronounced HOMA-S than those in the metformin group (mean treatment difference, 16.37; SD, 6.77; P < 0.05). There was a significant difference in the reduction of FPG between the two groups (-3.0 mmol/l vs. -2.8 mmol/l, p = 0.620 for pioglitazone and metformin, respectively).
Schernthaner et al.,2004 [10]	RCT	1194 patients (659 males and 535 females)	65, 146, and 339 patients were subjected to metformin dosages of 850mg, 1700mg, and 2550mg, respectively.	78 and 475 patients were subjected to 30mg and 45mg of pioglitazone, respectively.	52	The fasting insulin was significantly reduced in the pioglitazone group than in the metformin group (-2.4 µU/ml vs. 0 µU/ml, p<0.0001, respectively). The Mean change in the FPG was larger in patients receiving pioglitazone than in patients receiving metformin (-45.0±16.2 mg/dL vs. -39.6±16.2 mg/dL, respectively). 42 patients receiving pioglitazone and 39 receiving metformin were withdrawn from the study due to adverse events.
Fidan et al.,2011 [11]		40 patients (16 females and 24 males aged above 40 years)	Metformin was initiated at 850 mg dosage and adjusted to 3 x 850 mg at 10-day intervals	Rosiglitazone was initiated at 4mg and adjusted to 8 mg.	12	At the end of 3 months, the decrease in HOMA-IR was not significant for patients receiving metformin (from 4.5±3.0 to 3.4±3.5), while a significant difference was observed in the rosiglitazone group (from 4.8 ± 3.4 to 2.8 ± 2.5, p<0.05). An insignificant difference in the FPG was observed in the metformin group (from 144 ± 24mg/dL to 130 ± 36mg/dL), while a significant change was recorded in the rosiglitazone group (from 149 ± 30mg/dL to 116 ± 20mg/dL p<0.05).
Chu et al.,2002 [12]	RCT	22 patients (20 males and 2 females; mean age 56±2 years).	12 patients were initially subjected to receive 850mg daily	10 patients were initially subjected to 200mg troglitazone daily.	16	An insignificant change in the FPG was recorded between the metformin and troglitazone groups (-32mg/dL vs. -36mg/dL, p=0.90). Both groups’ fasting insulin levels decreased; however, the difference was insignificant (-6mU/l vs. -18mU/l, p=0.30, for metformin and troglitazone, respectively).
Hällsten et al.,2002 [13]	RCT	41 patients (28 males and 13 females)	13 patients were initially subjected to 500mg of metformin for 2 weeks and thereafter 1g daily	14 patients were initially subjected to 2mg of rosiglitazone for 2 weeks; thereafter, 4mg daily	26	A significant change in FPG was recorded in the metformin group (from 8.0±0.5mmol/l to 6.8±0.3mmol/l, p < 0.001), while an insignificant decrease in FPG was recorded in the rosiglitazone group (from 7.2±0.3 to 6.8±0.3mmol/l). The decrease in fasting serum insulin was insignificant in both metformin (from 11.7± 2.1mU/l to 8.8±1.1mU/l) and rosiglitazone group (from 8.6±1.5 to 6.6±0.4).
Kautzky-Willer et al.,2005 [14]	RCT	20 patients (14 males and 6 females)	9 patients were initially subjected to an 850mg metformin dose once every day for 1 week; thereafter, the dose was given twice daily.	11 patients were initially subjected to a 400mg daily dosage of troglitazone for 1 week; thereafter, the dose was adjusted to 600mg daily.	16	A significant improvement in fasting insulin resistance (HOMA) was recorded in the troglitazone group (from 5.3±0.9 to 3.4±0.7), while the change was insignificant for the metformin group (from 6.3±0.9 to 4.1±0.8, respectively).
Tiikkainen et al.,2004 [15]		20 patients (13 females and 7 males)	11 patients in the metformin group received 1g dosage for 16 weeks	9 patients were subjected to 4mg of rosiglitazone for 16 weeks	16	Fasting serum insulin decreased significantly in both groups; however, the statistical analysis showed that the difference was not significant (4±1 and 4±2mU/l for rosiglitazone and metformin groups, respectively) A significant decrease in FPG was recorded in rosiglitazone (from 8.8±0.8 to 7.3±0.4mmol/l) and metformin group (from 8.2±0.7 to 6.7±0.2mmol/l)
Ceriello et al.,2005 [16]	RCT	940 patients (382 females and 558 males)	195 patients were subjected to pioglitazone	187 patients received metformin	52	There was a statistically insignificant difference in the change of FPG between pioglitazone and metformin (-2.52±0.145 vs. -2.46±0.148mmol/l, respectively). Pioglitazone had a significantly higher reduction in fasting insulin than metformin (-12.77±2.40 vs. -4.59±2.44mmol/l).
Roden et al.,2005 [17]	RCT	1788 patients (1005 males and 783 females aged 35 – 75 years)	597 patients received metformin	597 patients received pioglitazone	52	A higher significant increase in insulin sensitivity was recorded among patients in the pioglitazone group than in the metformin group (p<0.001). A significantly higher reduction in fasting serum insulin was recorded among patients in the pioglitazone group (from −16.12±3.15 to −27.38 ± 2.17pmol/l).
Basu et al.,2008 [18]	RCT	31 patients (15 females and 16 males)	16 patients were subjected to 1000mg of metformin twice every day	15 patients were subjected to 45mg of pioglitazone daily	16	No significant change in fasting glucose was recorded in either patients receiving pioglitazone (from 157 ± 10 to 140 ± 13mg/dL) or metformin (from 148 ± 12 to 146 ± 10mg/dL).

Glucose Tolerance

**Figure 4:**
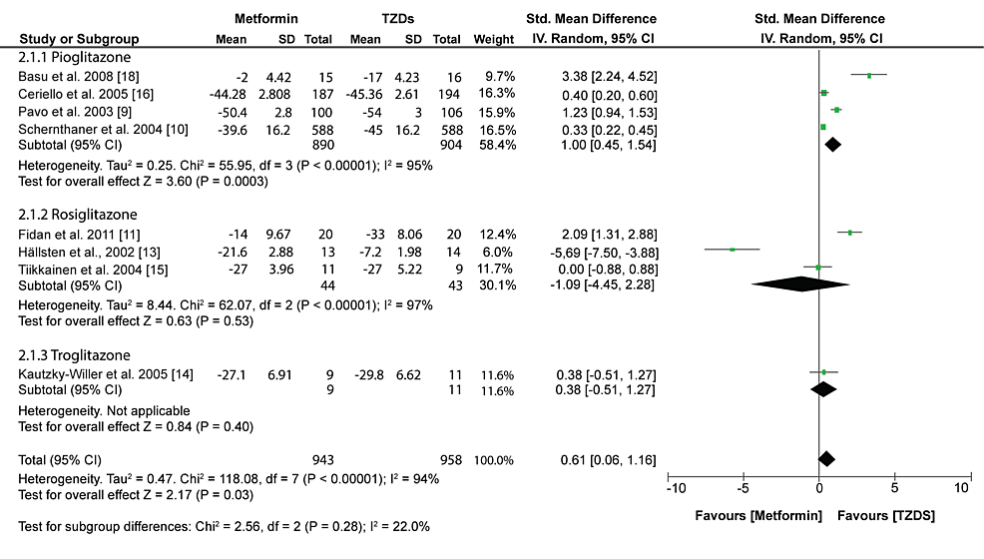
Forest plot comparing the effects of TZDs and Metformin on FPG Pavo et al.,2003 [9], Schernthaner et al.,2004 [10], Fidan et al.,2011 [11], Hällsten et al.,2002 [13], Kautzky-Willer et al.,2005 [14], Tiikkainen et al.,2004 [15], Ceriello et al.,2005 [16], Basu et al.,2008 [18]

According to the world health organization (WHO), individuals are described as glucose intolerant if they manifest either impaired fasting glucose (IFG) or impaired glucose tolerance (IGT) [19]. IFG is defined as FPG above 6.0mmol/l; therefore, a reduction in FPG would increase glucose tolerance among patients with T2DM. Our meta-analysis showed that TZDs significantly reduced the FPG more than Metformin (SMD:0.61; 95% CI:0.06, 1.16: p=0.03) (fig 4). 

However, a subgroup analysis showed varied results when different TZDs were used. Pioglitazone proved to have a significantly higher reduction in FPG than Metformin (SMD: 1.00; 95% CI: 0.45, 1.54; p = 0.0003). On the other hand, rosiglitazone and troglitazone had similar effect on FPG as metformin (SMD: -1.09; 95% CI: -4.45, 2.28; p = 0.53 and SMD: 0.38; 95% CI: -0.51, 1.27; p = 0.40, respectively). All FPG outcomes were converted to mg/dL for a uniform meta-analysis.

Insulin Sensitivity

Insulin sensitivity was calculated using the QUICKI formula, of which FPG outcomes were converted to mg/dL, and fasting serum insulin (FSI) outcomes were converted to µU/ml. Statistical analysis showed that both Metformin and TZDs significantly improved insulin sensitivity from baseline (0.292 ± 0.017 to 0.306 ± 0.019, p<0.00001 and 0.296 ± 0.019 to 0.316 ± 0.019, p<0.00001, respectively) (Table 2). However, a comparison showed that TZDs significantly improved insulin sensitivity more than metformin (p = 0.0003).

**Table 2:** Comparison of the effects of Metformin and TZDs on insulin sensitivity assessed with QUICKI Pavo et al.,2003 [9], Schernthaner et al.,2004 [10], Fidan et al.,2011 [11], Chu et al.,2002 [12], Hällsten et al.,2002 [13], Kautzky-Willer et al.,2005 [14], Tiikkainen et al.,2004 [15], Ceriello et al.,2005 [16], Roden et al.,2005 [17]

Author ID	Metformin		TZDs	
	QUCKI at Baseline	QUICKI at endpoint	QUCKI at Baseline	QUICKI at endpoint
Chu et al.,2002 [12]	0.256	0.269	0.257	0.277
Fidan et al.,2011 [11]	0.306	0.319	0.306	0.329
Hallsten et al.,2002 [13]	0.310	0.330	0.328	0.344
Kautzky-Willer et al.,2005 [14]	0.294	0.313	0.298	0.319
Pavo et al.,2003 [9]	0.278	0.289	0.287	0.307
Schernthaner et al.,2004 [10]	0.289	0.298	0.288	0.304
Ceriello et al.,2005 [16]	0.290	0.301	0.294	0.311
Tiikkainen et al.,2004 [15]	0.301	0.325	0.304	0.329
Roden et al.,2005 [17]	0.304	0.315	0.303	0.321
Average (mean ± SD)	0.292 ± 0.017	0.306 ± 0.019	0.296 ± 0.019	0.316 ± 0.019
Significance (p-value)	P < 0.00001		P < 0.00001	
Overall significance between Metformin and TZDs endpoints	P = 0.0003			

Glycemic Control

**Figure 5:**
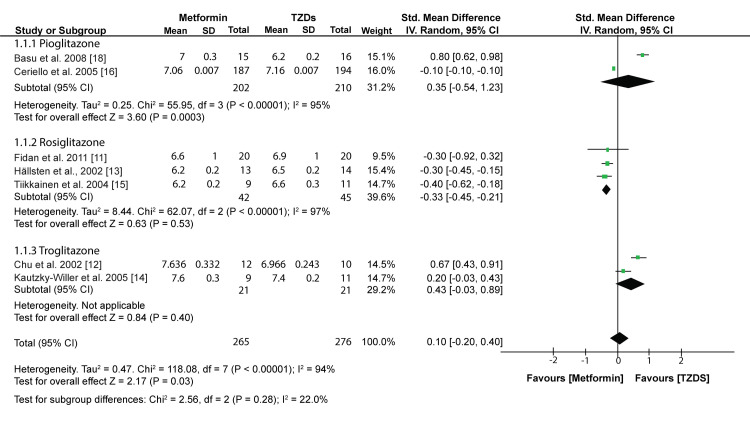
Forest plot comparing the effects of TZDs and Metformin on HBA1c Fidan et al.,2011 [11], Chu et al.,2002 [12], Hällsten et al.,2002 [13], Kautzky-Willer et al.,2005 [14], Tiikkainen et al.,2004 [15], Ceriello et al.,2005 [16], Basu et al.,2008 [18]

A meta-analysis of outcomes from 7 included studies showed that TZDs had a similar effect on the glycosylated hemoglobin (HBA1c) as metformin (MD: 0.10; 95% CI: -0.20, 0.40; p=0.52) (fig 5). 

However, a subgroup analysis showed varied results for different TZDs. Patients receiving Rosiglitazone had significantly lower reductions in HBA1c than those receiving Metformin (MD: -0.33; 95% CI: -0.45, -0.21; p<0.00001). On the other hand, patients receiving pioglitazone and troglitazone had similar HBA1c reductions as those receiving Metformin (MD: 0.35; 95% CI: -0.54, 1.23; p = 0.44 and MD: 0.43; 95% CI: -0.03, 0.89; p = 0.07).

Discussion

The current study compared the effect of TZDs and Metformin on insulin sensitivity and glucose tolerance among patients with T2DM. The meta-analysis shows that TZDs significantly improved in FPG than Metformin. Similarly, a statistical analysis showed that TZDs are better insulin-sensitizing drugs than Metformin. However, the pooled results show that Metformin and TZDs similarly affect glycosylated hemoglobin.

Even though our study shows that TZDs significantly improve FPG, a subgroup analysis has demonstrated that each TZD offers different results. Rosiglitazone showed to have a similar effect on FPG as Metformin. This is also evident in a previous randomized trial which showed that the 24 T2DM patients receiving Rosiglitazone had a similar decrease in the mean FPG as the 28 patients that received Metformin (-1.5 + 0.6 mmol/l, P < 0.005) and -1.6 + 0.6 mmol/l, P < 0.005, respectively) [20]. Similarly, Jung and colleagues reported that the 14 T2DM patients randomized to the 4mg/day rosiglitazone group had a similar effect on the reduction of FPG as the 13 patients randomized to the 500mg metformin group (8.8 + 2.3mmol/l vs. 9.1 + 1.6mmol/l at six months, respectively) [21]. That study says that the magnitude of FPG reduction was not optimal; however, the change was sufficient to show that the two insulin-sensitizing drugs came into effect. Another retrospective study on 250 patients with T2DM showed that as monotherapy, Rosiglitazone, and Metformin had a similar mean percentage change in FPG (34.0 ± 6.8% vs. 32.1 ± 7.2%, p= 0.945, respectively) [22].

Similarly, troglitazone seems to have the same effect as Metformin on reducing FPG. This outcome concurs with a previous randomized trial which reported that at the end of 3 months, monotherapy metformin and troglitazone significantly lowered the FPG concentration by 58mg/dL and 54mg/dL, respectively [23]. These changes accounted for about 20% of both groups. However, these results contradict Chu and colleagues, who reported that troglitazone had a significantly higher reduction in the FPG concentration than Metformin (32% and 36%, respectively; P < 0.001) [12]. The significant change in that study can be attributed to the fact that troglitazone was twice as effective as Metformin in the increment of insulin-stimulated glucose disposal.

On the other hand, pioglitazone has significantly reduced the FPG concentration than Metformin. This was also evident in a recently completed clinical trial which reported that after 3-month follow-up, patients that received pioglitazone had a significant reduction in the FPG concentration than patients that received Metformin (5.4 (1.2) vs. 6.5 (2.6), respectively; p<0.05) [24]. Contrary to these results, Sharma and colleagues reported that at the 12-week follow-up, Metformin and pioglitazone showed the same effect on the FPG concentration (6·4 ± 1·2 and 6·0 ± 1·4mmol/respectively=0.221) [25]. However, the study shows that pioglitazone was more effective in reducing the post-breakfast plasma glucose (PBPG) concentration than Metformin after the effect of gliclazide was discounted in both groups.

The other significant outcome analyzed in the current study was insulin sensitivity which can be measured using several simple surrogate indexes, including the homeostasis model assessment (HOMA), Quantitative insulin sensitivity check index (QUICKI), and Adipose Tissue Insulin Resistance Index. However, our study assessed insulin sensitivity using QUICKI due to the following advantages; First, many independent studies found that QUICKI has an excellent linear correlation to the glucose clamp estimates in obese, diabetes, hypertension, and healthy patients [26-29]. QUICKI is also considered effective for large clinical research studies to follow changes after therapeutic interventions and to evaluate insulin sensitivity in studies that were not a primary interest [30]. The statistical analysis carried out in the current study has shown that TZDs significantly improve insulin sensitivity more than Metformin. This result is supported by a recently completed clinical trial which reported that after three months follow-up period, the insulin sensitivity as measured using QUICKI was significantly improved among patients that received the TZD pioglitazone (0.59 (0.12) vs. 0.54(0.09), for pioglitazone and Metformin respectively; p<0.001) [24].

Similarly, Hällsten and colleagues reported that Rosiglitazone significantly improved whole-body insulin sensitivity (44% improvement) [13]. This improvement was attributed to the fact that Rosiglitazone significantly improved the glucose disposal rate more than Metformin. To the authors' surprise, Metformin did not show any change in insulin sensitivity despite significantly improving the glycemic control and reducing the body weight of T2DM patients.

Evidence in other studies using the HOMA assessment criteria has shown that TZDs significantly improve insulin sensitivity more than Metformin. For example, a double-blind randomized of 205 patients with recently diagnosed T2DM reported that after a 32-week follow-up, the HOMA-S was significantly improved by 14.9% in patients that received pioglitazone, while no change was observed for patients receiving Metformin. Further statistical analysis showed that pioglitazone significantly improved the HOMA-S more than Metformin (p = 0.020). Contradictory results were also noted in a study by Kautzky-Willer et al. [14], which showed that Metformin was more effective in incrementing dynamic insulin sensitivity than troglitazone. This contradiction can be attributed to the fact that the follow-up duration in that study was short such that the full effect of troglitazone on peripheral glucose uptake could not be achieved since, in dynamic stimulated conditions, it usually takes a longer period than metformin [31].

Additionally, improvement in insulin sensitivity was dependent on the decrease in the total insulin secretion, thus showing that insulin resistance poses less stress on the β-cells and proving that insulin sensitivity and secretion are highly related [32]. Fidan and colleagues also assessed insulin sensitivity using the HOMA-IR and found that in the third month, Metformin had no significant effect on the HOMA-IR, while Rosiglitazone significantly improved the HOMA-IR (p<0.05). Further statistical analysis showed that the difference in HOMA-IR between the two treatment groups was insignificant (3.4 ± 3.5 vs. 2.8 ± 2.5 for Metformin and Rosiglitazone, respectively; NS).

With the significant change in the FPG levels, one would expect that the HBA1c would be significantly different; however, a meta-analysis of outcomes from 7 included studies showed that the effect on glycemic control (HBA1c) was similar between the TZDs and Metformin. For patients with T2DM, the glycemic control, which is reflected by a decrease in HBA1c, is usually the sum of changes in numerous variables that affect the glucose metabolism in fasting and postprandial state. The two most possible explanation for this insignificant difference is that the patients reported in each study represented a different subset of patients, and some of the studies were designed to demonstrate the non-inferiority of the drug therapies which they were able to establish. However, our results concur with the observations made by Vilar et al. [22], which were as follows; patients randomized to the rosiglitazone and metformin group had similar mean reductions in HBA1c (p = 0.088), and the rate of patients achieving <7% HBA1c was not significant in either group (p = 0.956). Jung and colleagues also showed that the HBA1c levels were significantly improved from baseline in both Rosiglitazone (p<0.01) and Metformin (p<0.05) groups; however, further analysis shows that the change in the two groups was statistically insignificant [21]. A previous systematic review also seems to support these results as it claims that TZDs and Metformin exert the same improvements in glycemic control, of which values of up to 20% improvement in HBA1c have been observed after 3 to six months of treatment with either drug [33]. Contradictory results were also established in a randomized trial which showed that Metformin had a slightly improved HBA1c than pioglitazone [18]. The change in that study was assumed to occur because metformin insulin action was lower during treatment. It is also important to note that among patients with T2DM, dietary therapies may be considered to improve glycemic control. Our most recent meta-analyses established that ketogenic diets significantly improve glycemic control in T2DM patients [34,35].

The current study's design did not allow us to carry out a meta-analysis on weight control, but it is important to note that a decrease in insulin sensitivity among patients with T2DM is also associated with weight gain; therefore, weight control is important when treating these patients. However, studies have shown that the weight gain observed in T2DM patients receiving TZDs, especially pioglitazone, is associated with improved insulin sensitivity and glycemic control [36,37]. Another previous study showed that patients that received TZDs had a redistribution of adipose tissue with an increase in the subcutaneous fat and visceral fat decrease [38]. Due to this shift from visceral adipose deposition to subcutaneous, TZDs can increase insulin sensitivity. Other studies have shown that TZDs also reduce ectopic myocellular fat, which is strongly correlated to the increase in insulin sensitivity.

Evidence also seems to support the hypothesis that combination therapy may improve the therapeutic effects of the drugs. A randomized trial by Fonseca and colleagues showed that the FPG levels did not change significantly in patients receiving Metformin only. Still, patients that received the combination therapy (Metformin plus Rosiglitazone) had a significant change in their FPG levels (p<0.0001) [39]. The study also showed that when Rosiglitazone was added to the maximum metformin doses, the insulin sensitivity assessed using the HOMA-S scores was significantly improved (An improvement of 1.7 units and 3.8 units in the 4mg/dL and 8mg/dL rosiglitazone groups, respectively). Further analysis showed that adding Rosiglitazone to Metformin significantly reduced the HBA1c levels (0.56% and 0.78% for 4mg/dL and 8mg/dL groups, respectively). A significant increase in HBA1c levels was observed in the metformin group (0.45%). Triple combination therapy of Metformin, TZDs, and insulin also seems to impact glycemic control and insulin sensitivity significantly. Home and colleagues reported that at the 24th week, patients that received the triple therapy (Metformin, Rosiglitazone, and insulin) had a significant improvement in the HBA1c levels than those treated with only insulin (difference −0.7 (−0.8, −0.5) %, P < 0.001) [40]. Moreover, the rate of patients achieving HBA1c levels of <=6.5 and <7.0% was significantly higher in the triple therapy than in the control group. The FPG levels were also significantly reduced in the combination therapy group than the control group (−1.4 (−1.9, −0.9) mmol/l, P < 0.001). Another randomized trial that evaluated the efficacy of triple therapy (Metformin, Rosiglitazone, and insulin) among 16 obese patients with T2DM reported that the triple therapy had a significant improvement in the HBA1c levels from the baseline (P = 0.004) [41]. In fact, by the end of the trial, patients that had received the combination therapy had achieved HBA1c levels that were closer to the reference value (4.4 - 6.4%) and were 2.0% points better than patients that received insulin only (p<0.001). The study also showed that when the insulin sensitivity was assessed using the "tracer technique," the combination therapy patients were found to have improved. The improvement in insulin sensitivity was associated with the increase in glucose oxidation and not glucose storage.

When managing T2DM patients, safety is also an important outcome that must be considered. Troglitazone, a TZD used as an insulin-sensitizing drug for patients with T2DM, has been withdrawn in some markets, such as the United States, due to reports that the drug is associated with liver toxicity [42]. On the other hand, some studies have shown that Rosiglitazone may be associated with cardiovascular events. A previous systematic review and meta-analysis of 42 trials carried out in 2007 showed that Rosiglitazone was associated with increased myocardial infarction (OR) = 1.43, 95% CI = 1.03-1.98, p = 0.03) and cardiovascular deaths (OR = 1.64, 95% CI = 0.98-2.74, p = 0.060) [43]. In the same year, another meta-analysis showed that Rosiglitazone was associated with increased myocardial infarction (RR = 1.42, 95% CI = 1.06-1.93, p = 0.02) and heart failures (RR = 2.09, 95% CI = 1.52-2.88, p < 0.001) [44]. However, in that study, mortality from cardiovascular events was not increased (RR = 0.90, p = 0.53). These publications forced the United States Food and Drug Administration (FDA) to review the safety concerns of Rosiglitazone, of which the convened review board voted 20:3 that evidence suggested Rosiglitazone was associated with increased risk of cardiovascular events and 20:1 that the overall risk-benefit ratio of the drug justified its continued use [45,46]. In 2010 the European Marketing Authority recommended the removal of Rosiglitazone from the market despite the 2000 RECORD study showing no difference in the cardiovascular events or deaths between rosiglitazone, Metformin, or sulfonylurea treatments [47,48]. Similarly, studies published after 2010 have shown that Rosiglitazone has shown no cardiovascular mortality or morbidity. There is no evidence suggesting that pioglitazone is associated with increased cardiovascular risks. In fact, a previous meta-analysis of 3 randomized trials, including 4980 patients with insulin resistance, prediabetes, and diabetes, reported that pioglitazone was associated with lower risks of recurrent stroke (HR = 0.68, p = 0.01) and future major vascular events (HR = 0.75, p = 0.0001) [49]. The review also shows that pioglitazone did not affect all-cause mortality or heart failure.

Limitations

The current review was subject to several limitations, including a high heterogeneity observed in the meta-analysis of outcomes related to glycemic control. This heterogeneity can be attributed to the fact that each study used varying dosages, and some had concise follow-up periods. However, the heterogeneity did not influence the results considering that most studies included in the analysis were randomized trials with minimum publication bias. The study also included TZDs such as Rosiglitazone and troglitazone, which may have been withdrawn in several markets. However, given the design of this study was not to evaluate the safety, the drugs were used to inform on the effects of TZDs on insulin sensitivity and glucose tolerance. The other limitation was associated with the eligibility criteria of this systematic review which only allowed the inclusion of articles published in English. This criterion led to excluding articles related to our topic but written in other languages that could have been used to inform our scientific research and improve this systematic review's statistical power.

## Conclusions

The glucose intolerance and insulin resistance in patients with T2DM are addressed using various therapies, including dietary plans, and oral agents such as TZDs, Metformin, and insulin. The evidence in our current study has shown that TZDs are better at reducing FPG levels and improving insulin sensitivity than Metformin. However, the analysis has demonstrated that Metformin and TZDs have a similar effect on improving glycemic control as assessed using the HBA1c levels. From this analysis, we can suggest that TZDs have better long-term benefits in patients with T2DM. However, safety is critical, and health professionals should sufficiently monitor the patients administered the TZDs. We also recommend that future studies carry out extensive clinical trials to identify the association between TZDs such as Rosiglitazone and pioglitazone on cardiovascular events.
